# Enhancing radiation response by a second-generation TRAIL receptor agonist using a new in vitro organoid model system

**DOI:** 10.1016/j.ctro.2020.05.012

**Published:** 2020-06-09

**Authors:** Shuraila F. Zerp, Zainab Bibi, Inge Verbrugge, Emile E. Voest, Marcel Verheij

**Affiliations:** a Division of Cell Biology, The Netherlands Cancer Institute – Antoni van Leeuwenhoek Hospital, Amsterdam, The Netherlands; b Division of Tumor Biology and Immunology, The Netherlands Cancer Institute – Antoni van Leeuwenhoek Hospital, Amsterdam, The Netherlands; c Division of Oncogenomics, The Netherlands Cancer Institute – Antoni van Leeuwenhoek Hospital, Amsterdam, The Netherlands; d Department of Radiation Oncology, The Netherlands Cancer Institute – Antoni van Leeuwenhoek Hospital, Amsterdam, The Netherlands

**Keywords:** TRAIL receptor agonist, Radiation, Clonogenic survival, Organoids, Colorectal carcinoma

## Abstract

**Background:**

For many cancer types, including colorectal carcinoma (CRC), combined modality treatments have shown to improve outcome, but are frequently associated with significant toxicity, illustrating the need for new therapeutic approaches. Based on preclinical data, TRAIL receptor agonists appeared to be promising agents for cancer therapy especially in combination with DNA damaging regimens. Here, we present the combination of the second-generation TRAIL receptor agonist APG-880 with radiation in a new and clinically relevant 3D model system.

**Methods:**

To investigate the effect of APG-880 in combination with radiation we performed short-term cytotoxicity and long-term clonogenic survival assays in established CRC cell lines, and in tumor organoids derived from colon cancer patients.

**Results:**

APG-880 is a potent inducer of apoptosis in CRC cell lines and in patient-derived CRC organoids. Furthermore, a supra-additive effect on cytotoxicity was found when APG-880 and radiation were combined simultaneously, with combination indices around 0.7. Lastly, in the long-term survival assays, we demonstrated a radiosensitizing effect of APG-880 with dose enhancement factors between 1.3 and 1.5.

**Conclusions:**

In a new, clinically relevant CRC-organoid model system we demonstrated a more than additive combined effect between the second-generation TRAIL receptor agonist APG-880 and radiation.

## Highlights

• We evaluated the effect of the second-generation TRAIL receptor agonist APG-880 on radiation-induced cytotoxicity.
• The combined effect was studied in short-term and long-term cytotoxicity assays in established CRC cell lines, and tumor organoids derived from colon cancer patients.
• We observed a supra-additive effect on cytotoxicity when APG-880 and radiation were combined simultaneously, with combination indices around 0.7.
• In long-term survival assays, we demonstrated a radiosensitizing effect of APG-880 with dose enhancement factors between 1.3 and 1.5.

## 1 Introduction

Over 50% of all cancer patients receive radiation therapy during the course of their disease and radiotherapy contributes to approximately 40% of all cancer cures [1].

Colorectal carcinoma (CRC) is the third most common cancer worldwide with over one million new diagnoses per year and is expected to increase by 60% to more than 2.2 million new cases and 1.1 million deaths by 2030. The highest incidences occur in Western countries [2], [3]. Neoadjuvant (chemo)radiation is one of the standard treatment options for locally advanced CRC, though is associated with significant acute toxicity and surgical morbidity [4]. Therefore, there is a strong clinical need to develop novel combinations to improve the therapeutic ratio.

Radiosensitizers are agents that increase the lethal effect of ionizing radiation and, ideally, achieve greater tumor cytotoxicity than would have been expected from the additive effect of each modality. Some radiosensitizers are used in the clinic already, while others are being studied for new indications. For example, non-specific agents such as cisplatin or 5-FU have shown to improve local control and overall survival when added to radiation therapy and are integrated in standard treatment protocols [5], [6]. Based on the hallmarks of cancer, target-specific radiosensitizers have become available for clinical use, e.g. the EGFR-antagonist cetuximab [7] that reduces proliferative signaling. Others are still in early clinical development such as PARP inhibitors that interfere with radiation-induced DNA damage repair [8].

Since apoptosis is an important mechanism of radiation induced cell death, modulation of apoptosis sensitivity has been shown to increase tumor cell kill and relatively spare normal tissues, thereby enhancing therapeutic outcome [1], [9], [10]. Here, we focus on modulation of apoptosis by the tumor necrosis factor-related apoptosis-inducing ligand TRAIL. TRAIL is a homotrimeric cytokine expressed by immune cells and plays a protective role in immune mediated tumor surveillance [11], [12]. In human, TRAIL signals for apoptosis via TRAIL-receptor (TRAIL-R) 1 and TRAIL-R2, also known as Death Receptor 4 (DR4) and Death Receptor 5 (DR5), respectively. TRAIL induces clustering of DR4 and DR5, and can subsequently activate apoptotic pathways independently of mitochondrial involvement and p53-status. TRAIL acts preferentially on tumor cells and is non-toxic to most healthy tissue [13], [14]. TRAIL can also bind ‘decoy receptors’ DCR1 and DCR2 which lack functional death domains [15], [16] and a soluble receptor called osteoprotegerin [17].

A novel, second-generation TRAIL-receptor agonist has been engineered that simultaneously binds up to six TRAIL receptors, thereby improving the ability to form receptor clustering on cancer cells to induce superior anti-tumor efficacy *in vivo*, which is independent of cross-linking to Fc receptors [18]. The lead molecule, known as APG-880/ABBV-621 is currently being tested in phase I clinical trials (NCT03082209) [19].

The rational for combining TRAIL and radiation in our study, is that these agents activate the two distinct pathways leading to apoptosis [20]. Death receptor ligands like TRAIL, initiate apoptotic signaling via the extrinsic pathway [21], whereas radiation and other DNA damaging agents activate the intrinsic pathway to cell death. Simultaneous activation of both pathways has been shown to be particularly effective in inducing cell death [22], [23], [24]. Via active caspase 8, TRAIL receptor signaling can also trigger the intrinsic mitochondrial pathway through Bid cleavage into truncated Bid (tBid). tBid subsequently translocates to the mitochondria, leading to mitochondrial permeabilization and cytochrome *C* release [25].

Unfortunately, TRAIL-based therapies have not yet led to improved clinical responses mainly because the first-generation TRAIL receptor agonists (TRAs) failed efficacy and did not meet clinical expectations as tested in phase I and II trials [21], [26], [27]. For instance, TRAs dulanermin, mapatumumab, lexatumumab, conatumumab, tigatuzumab or drozitumab, as single agent or in combination studies did not lead to statistically significant anticancer activity in randomized controlled trials [28], [29]. There are several factors that have contributed to this apparent translational failure. First, the extent by which first generation TRAIL molecules were able to bind and cluster their receptors, and subsequently activate the extrinsic pathway has been limited. This was most likely due to the bivalent nature of antibodies that allows crosslinking of only two DRs leading to inefficient DISC formation [28]. APG-880 induces better hexavalent clustering of TRAIL receptors, and furthermore does not require Fcγ-R-mediated crosslinking for optimal *in vivo* efficacy suggesting that this second generation molecule may be superior to previously tested TRAs [19].

Second, the model systems used over the past years to test TRAIL efficacy were suboptimal in their capacity to predict clinical activity. Indeed, 2D cell culturing techniques are now considered structurally and functionally inferior to mimic cancer and are of limited use to predict successful clinical translation [30]. Tumor-derived organoids have the potential to serve as a pre-clinical model [31] and to better predict treatment response of individual patients. As TRAIL-receptors are commonly expressed in colorectal tumor tissue [32], we evaluated the efficacy of APG-880 alone and in combination with radiation therapy both in CRC cell lines, and in patient-derived CRC-organoids.

## 2 Materials & methods

### 2.1 Reagents

APG-880 stock solution (10.6 mg/ml) was provided by AbbVie (North Chicago, IL, USA), aliquoted in 2 μl portions and stored at −80 °C. Thawed samples were only used once.

### 2.2 Cell culture

Colon cancer derived cell lines HCT116 and HT29 were purchased from the American Type Culture Collection (ATCC). Jurkat cell line J16, were kindly provided by prof. dr. J. Borst (The Netherlands Cancer Institute, Amsterdam). All cell lines were grown according to ATCC protocols.

### 2.3 Organoid culture

Surgical specimens (in case of organoid cultures ITO17 and ITO60), or core needle biopsy material (in case of organoid culture ITO77), collected within a clinical trial at the NKI (NL48824.031.14) were dissected, stored in the central biobank and used for the establishment of organoids. For our experiments we used three different biobank-stored organoids from three different patients. After institutional approval, the biobank stored organoids ITO17, ITO60 and ITO77 were thawed and cultured according to the protocol earlier described [33]. In short, organoids were grown in Geltrex LDEV-Free hESC-qualified Reduced Growth Factor Basement Membrane Matrix (Life technologies, #A1413202 Carlsbad, CA, USA) and covered in the appropriate volume of growth medium Advanced DMEM/F-12 (Life Technologies, cat. no. 12634-010) supplemented with 2 mM GlutaMAX (Invitrogen #35050-079, 10 mM HEPES Invitrogen #15630-056, 100 units/ml and 100 mg/ml of Penicillin/Streptomycin, respectively (Invitrogen #15140-122), 10% Noggin conditioned medium, 20% R-spondin1 conditioned medium, B27 supplement (Invitrogen #17504-044, 1.25 mM N-Acetylcysteine (Sigma-Aldrich #A9165-5G), 50 ng/ml human recombinant EGF (BD bioscience #354052) 10 mM Nicotinamide Sigma-Aldrich #N0636), 500 nM A-83-01 Tocris #2939), 3 μM SB202190 Cayman Chemicals #10010399), 10 μM Prostaglandin E2 (Cayman Chemicals #14010-1) 10uM Y-27632 Sigma-Aldrich #Y0503) [34], [35], [36].

Regarding the organoids used in this study, organoid ITO77 was derived from a peritoneal metastasis and organoids ITO17 and ITO60 originated from a primary colorectal carcinoma. Their mismatch repair (MMR) status is known: ITO17 and ITO60 are MMR proficient, while ITO77 is MMR deficient.

### 2.4 In vitro irradiation procedure

Cells were exposed to gamma rays from a Gammacell® 40 Exactor (Best Theratronics Ltd. Ottawa, Ontario Canada) at a dose rate of approximately 1 Gy/min.

### 2.5 Apoptosis analysis

Apoptosis was determined by staining cells with bis-benzimide or propidium iodide to detect morphological nuclear changes as described earlier [37] or FACScan analysis as described earlier [38]. A third method of apoptosis detection was performed with the IncuCyte Live-Cell Analysis System. In this assay the activation of caspase-3 or caspase-7 was measured by an inert non-fluorescent DEVD (Asp-Glu-Val-Asp) peptide motif substrate that is able to cross the cell membrane and can be cleaved by activated caspase-3/7 to release a green DNA-binding fluorescent label. In order to detect apoptosis, 5 μM of the caspase-3/7 reagent (IncuCyte, Essen BioScience #4440, Michigan, USA) was added to 96 well culture plates. Cells were in a proliferating state prior to APG-880 and/or radiation treatment. Images (10x amplification) of the cells with fluorescently-labeled nuclei were captured every 4 h in the IncuCyte® ZOOM System. Subsequent analysis was performed with the IncuCyte® ZOOM System software.

### 2.6 Western blotting

Western blot analysis was performed to detect DR4 and DR5 protein. Cells were treated with radiation and/or APG-880, washed and lysed in Triton lysis buffer as described earlier [37]. Blots were probed with an anti-DR4 rabbit polyclonal antibody C-terminus (#AB16955 Burlington, MA, USA) and anti-DR5 antibody (TNFRSF10B Mouse anti-human TRAIL R2 clone B-D37 monoclonal antibody cellsciences #CDM237 Newburyport, MA, USA) in 5% Nutrilon in TBS-T. After secondary horseradish peroxidase-conjugated antibody incubation, proteins were detected using the ChemiDoc™ Imaging Systems.

### 2.7 Epitope expression by FACS

DR4 and DR5 epitope expression was determined by flow cytometry as described by Verbrugge et al. [22], using rabbit polyclonal anti-DR4 antibody or mouse anti-human TRAIL R2/DR5 monoclonal antibody, unconjugated, Clone B-D37.

### 2.8 Metabolic activity assay

To determine the cytotoxic potential of APG-880 in organoids we dissociated the organoids with TriplE Express (Invitrogen, #12605 Carlsbad, CA, USA) at day 1. We expanded the cells to form small organoids of comparable sizes, and treated them with a defined concentration range at day 4, then performed metabolic activity assays with the CellTiter‐Glo® 3D reagent kit (Promega, G9681, Madison, WI 53711 USA) at day 10.

### 2.9 Clonogenic survival assays

Single cells were plated and allowed to attach before treatment. Cells were irradiated and treated with the indicated amount of APG-880, or sham-treated, immediately after irradiation and cultured for at least 14 days to allow colony formation. Colonies were fixed and stained with 0.2% crystal violet/2.5% glutaraldehyde. Colonies consisting of 50 cells or more were counted. The surviving fraction of cells was calculated by normalizing plating efficiency values of the treated samples to the untreated controls.

### 2.10 Organoid survival assay

In order to determine the clonogenic potential of organoids we dissociated growing organoids with TriplE Express (Invitrogen, #12605 Carlsbad, CA, USA) at day 1. We plated the organoid derived single cells in Geltrex LDEV-Free hESC-qualified Reduced Growth Factor Basement Membrane Matrix (Life technologies, #A1413202 Carlsbad, CA, USA) and covered the matrix with growth medium as described above. Immediately after seeding the single cell suspension of the organoids, they were treated with the appropriate dose of radiation and/or concentration APG-880 and cultured for 14 days. Pictures of the colonies were taken with a cooled Hamamatsu ORCA R2 Black and White CCD-camera on a Zeiss AxioObserver microscope with a 10x/0.30 ECPlan-Neofluar Ph1 objective, run by ZEN2.3 Zeiss acquisition software. Analysis of the pictures and counting the number of organoids sized at least 50 cubic micrometer was performed with ImageJ. Organoids counts were done manually and double checked by an independent technician, in a blinded setting.

### 2.11 Combination index

The effects on cancer cells by ionizing radiation and APG-880 was characterized by calculating the Combination Index (CI) according to the classic isobologram equation described by Chou and Talalay [39]: CI = (D)_1_/(D_x_)_1_ + (D)_2_/(D_x_)_2,_ and was previously applied by Zerp et al. [38]. The Combination Index can either indicate additivity (CI = 1), synergism (CI < 1) or antagonism (CI > 1).

## 3 Results

### 3.1 DR4 and DR5 are expressed by colon cancer cell lines and colon cancer derived organoids

**Fig. 1:**
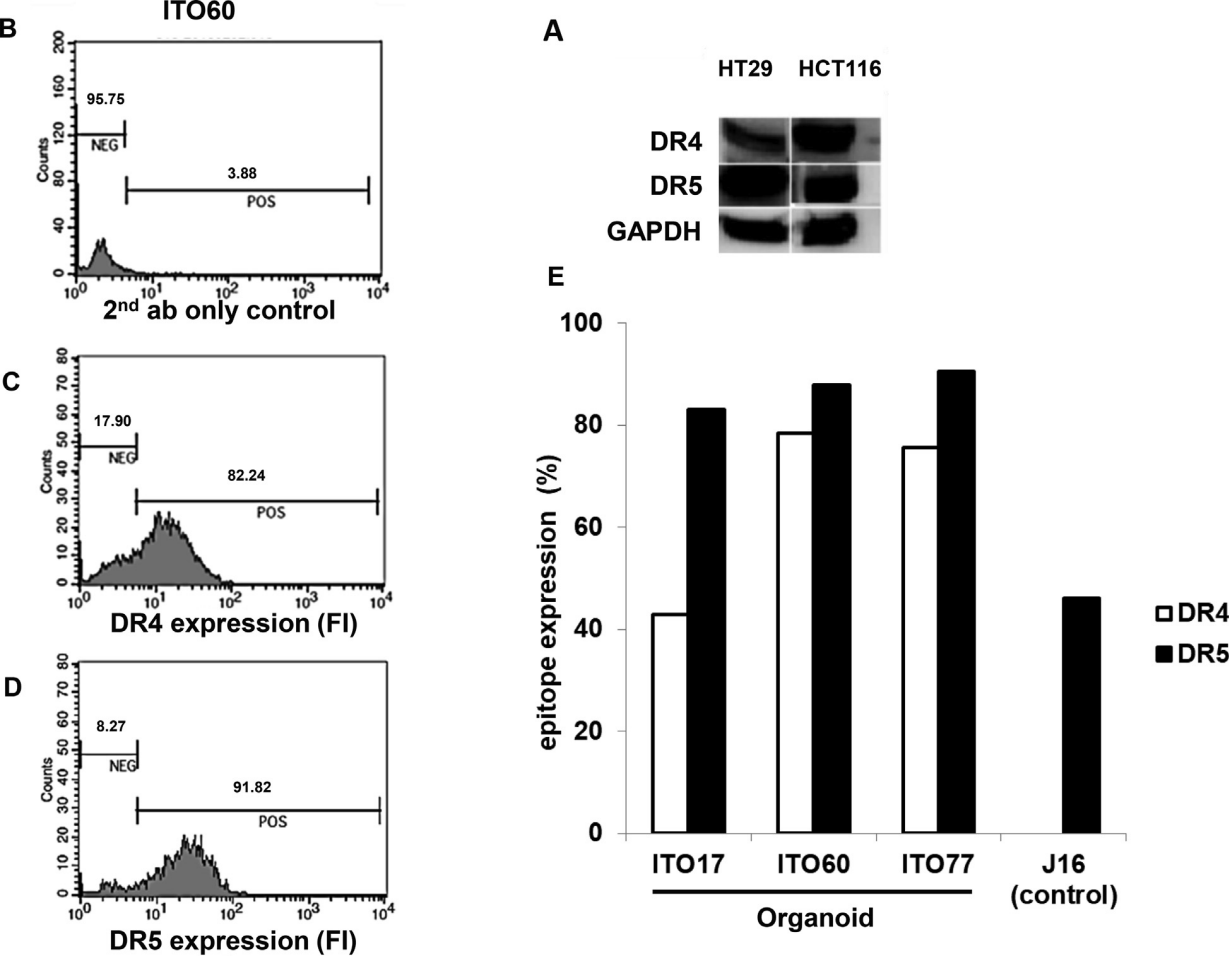
*CRC cell lines and organoids express DR4 and DR5.* Western blot analysis of DR4 and DR5 expression in HT29 and HCT116 cells (A). In (B), (C) and (D), CRC organoids express cell surface DR4 and DR5. Endogenous DR4 and DR5 expression on the cell surface of three different colorectal carcinoma patient derived organoids, ITO17, ITO60 and ITO77 analyzed by live-cell flow cytometry. (B) Negative control of organoid ITO60 consisting of secondary antibody only, in (C) anti-DR4 fluorescent staining of ITO60 and in (D) anti DR5 fluorescent staining of ITO60. (E) Quantification of the percentage DR4 and DR5 positive cells in the different organoids analyzed. J16 cells serve as a negative control for anti-DR4 as well as a positive control for anti-DR5.

First, we validated by Western blotting that DR4 and DR5 are expressed in HCT116 and HT-29 cell lines (Fig. 1A). Patient-derived CRC organoids also expressed surface DR4 and DR5, as determined by flow cytometry (Fig. 1B–E).

### 3.2 Colon cancer cell lines and colon cancer derived organoids undergo apoptosis upon APG-880 and radiation treatment

**Fig. 2:**
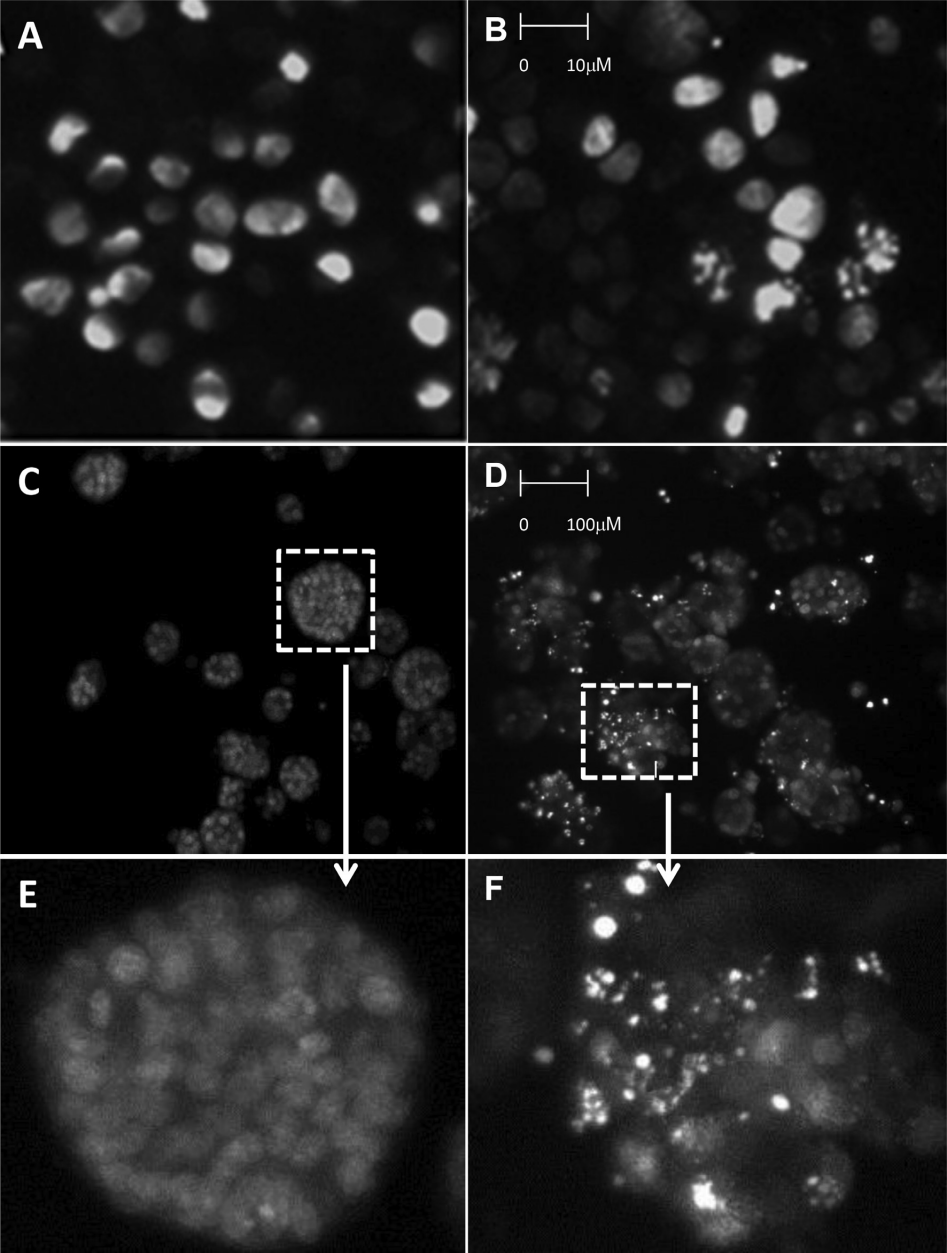
*APG-880 induces DNA fragmentation.* Staining with Hoechst demonstrated typical apoptotic body formation visualized by fluorescence microscopy. (A) Nuclei of HT29 control cells. (B) HT29 cells treated with 100 ng/ml APG-880. (C) Organoids ITO60 control. (D) Organoid ITO60 treated with 100 ng/ml APG-880. (E) and (F) Magnification of a cutout of respectively (C) and (D).

**Fig. 3:**
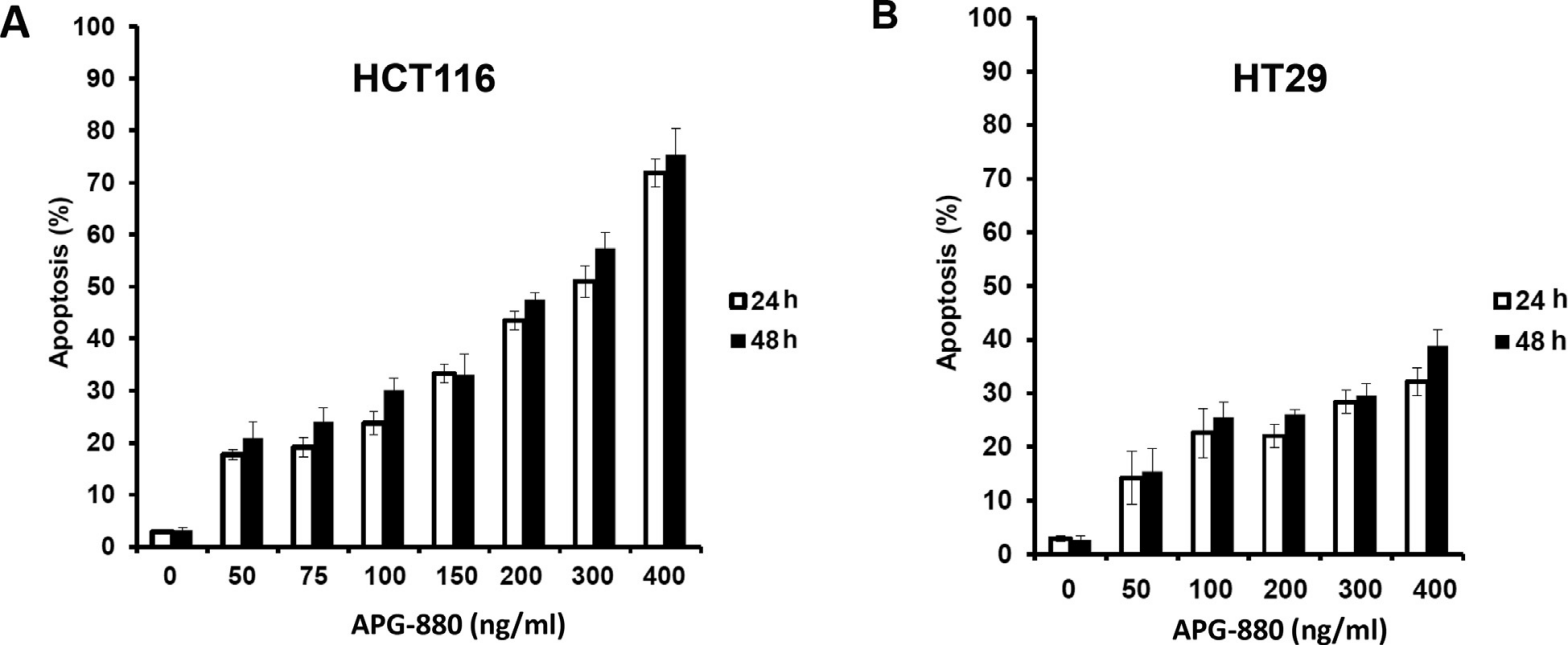
*APG-880 induces apoptosis in a dose-dependent manner in CRC cell lines*. Apoptosis induction by APG-880 HCT116 (A) and HT29 (B) as read-out by counting cells displaying DNA fragmentation.

**Fig. 4:**
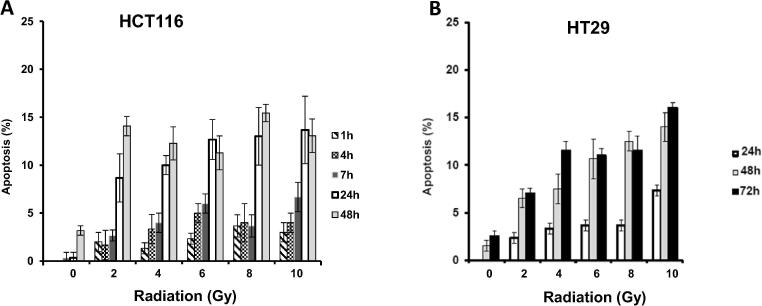
*Radiation induces apoptosis in a dose-dependent manner in CRC cell lines*. Apoptosis induction at 1, 4, 7, 24, 48 h after radiation in HCT116 (A) and 24, 48, 72 h after radiation in HT29 (B).

To confirm the apoptotic mode of cell death we first validated by Hoechst staining that APG-880 induces DNA condensation/fragmentation, a hallmark of apoptosis in HCT116 and HT29 cell lines and in CRC organoids (Fig. 2). To quantify apoptosis induction, HCT116 and HT29 cell lines were treated with APG-880 and apoptotic cells were counted (Fig. 3). This revealed a dose-dependent induction of apoptosis by APG-880 and higher sensitivity of HCT116 as compared to HT29 cells at both 24 and 48 h (Fig. 3). In these same cell lines we observed both a time- and dose-dependent induction of apoptosis by radiation (Fig. 4). Compared to HT29 cells, HCT116 cells showed an earlier onset of apoptosis induction with no further increase beyond 24–48 h.

Due to the 3D nature of the organoids we used a different quantification method that is presented with the results of the combination experiments in Section 3.5.

### 3.3 APG-880 and radiation decrease clonogenic cell survival

**Fig. 5:**
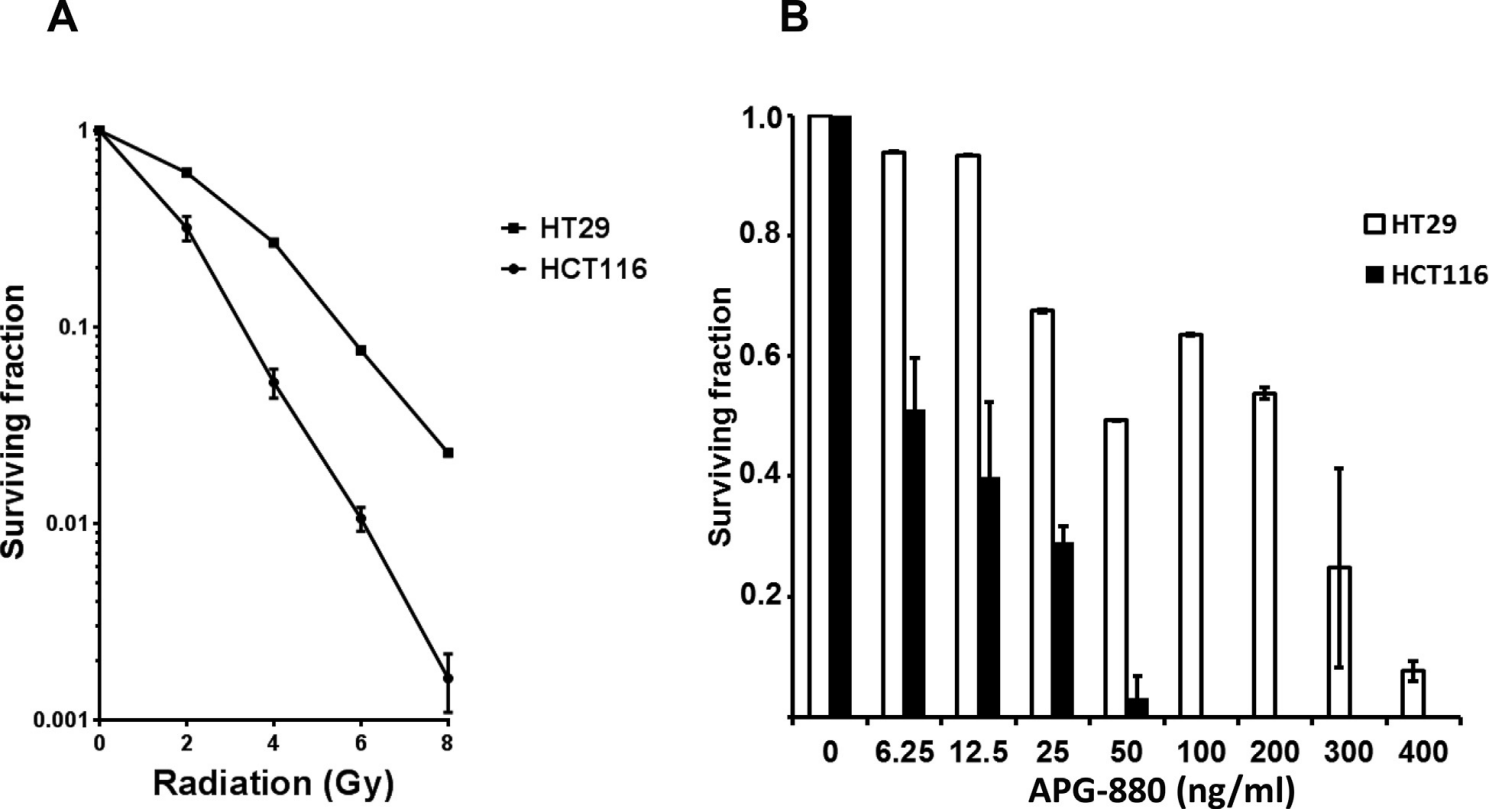
*APG-880 and radiation decrease clonogenic survival in cell lines*. (A) HCT116 (spheres) and HT29 (squares) clonogenic survival curves following increases doses of radiation (n = 3–4 experiments, performed in triplicate), error bars represent SEM. (B) Clonogenic survival graph of colon carcinoma cell lines HT29 and HCT116 upon APG-880 treatment. Graph shows the results of 1–3 experiments in triplicate, error bars represent SD.

We next established the sensitivity of the colon carcinoma derived cell lines HCT116 and HT29 towards radiation and APG-880 in clonogenic survival assays, the gold standard for long-term cytotoxicity. HCT116 was more sensitive than HT29 for both treatment modalities (Fig. 5A and B).

### 3.4 Colon cancer cell lines show a more than additive induction of cell death when exposed to the combination of radiation and APG-880

**Fig. 6:**
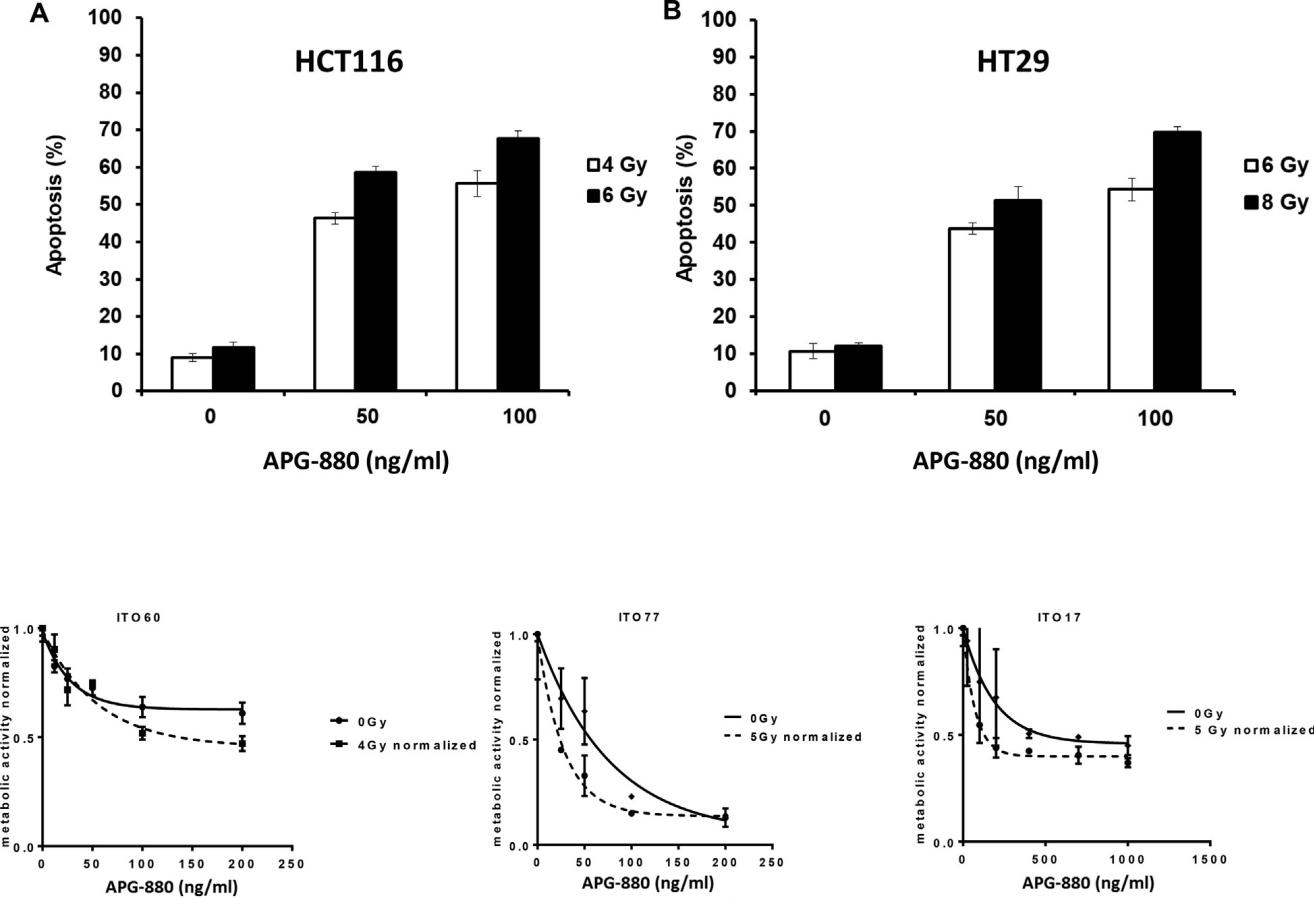
*Colon cancer cell lines show a more than additive effect when exposed to the combination of radiation and APG-880.* In graphs (A) and (B) combination experiments are shown where radiation and APG-880 were applied concurrently and apoptosis was determined after 24 h for HCT116 and 48 h for HT29. Data represent mean values ± SEM of an average of 3 independent experiments, performed in duplicate. Combination indices were calculated from these graphs. (C), (D), (E): Growing organoids were exposed to APG-880 for 6 days. Graph represents the average of 2 independent experiments in triplicate, error bars represent SEM.

When both treatment modalities were combined, we found a more than additive effect as quantified by calculating the combination index (CI) (Fig. 6A and B). For HCT116 13% apoptosis was the maximum percentage that could be achieved with radiation alone at t = 24 h. For HT29 13% apoptosis was the maximum for radiation at t = 48 h. Therefore, the CI’s calculated for instance at 13% were 0.78 for HCT116 at 24 h and 0.67 for HT29 at 48 h.

### 3.5 APG-880 enhances radiation responses in organoids

Because the 3D nature of the organoids hampered the Hoechst quantification method used in the cell lines, we applied a different quantification method for cell death/loss of viability, i.e. the cell titer glow 3D assay that is especially developed for measuring cell death in 3D cell culturing conditions (Fig. 6C). The cell titer glow 3D method showed a dose dependent cytotoxicity in three organoid lines tested towards both APG-880 and radiation. Furthermore, and consistent with the results in the cell lines we show in these organoids that within a limited concentration range, i.e. 50–100 ng/ml, combined treatment with APG-880 and radiation is more effective than treatment by radiation or APG-880 alone (Fig. 6C).

### 3.6 Colon cancer derived cell lines and organoids show enhanced reduction in clonogenic survival when exposed to the combination of radiation and APG-880

**Fig. 7:**
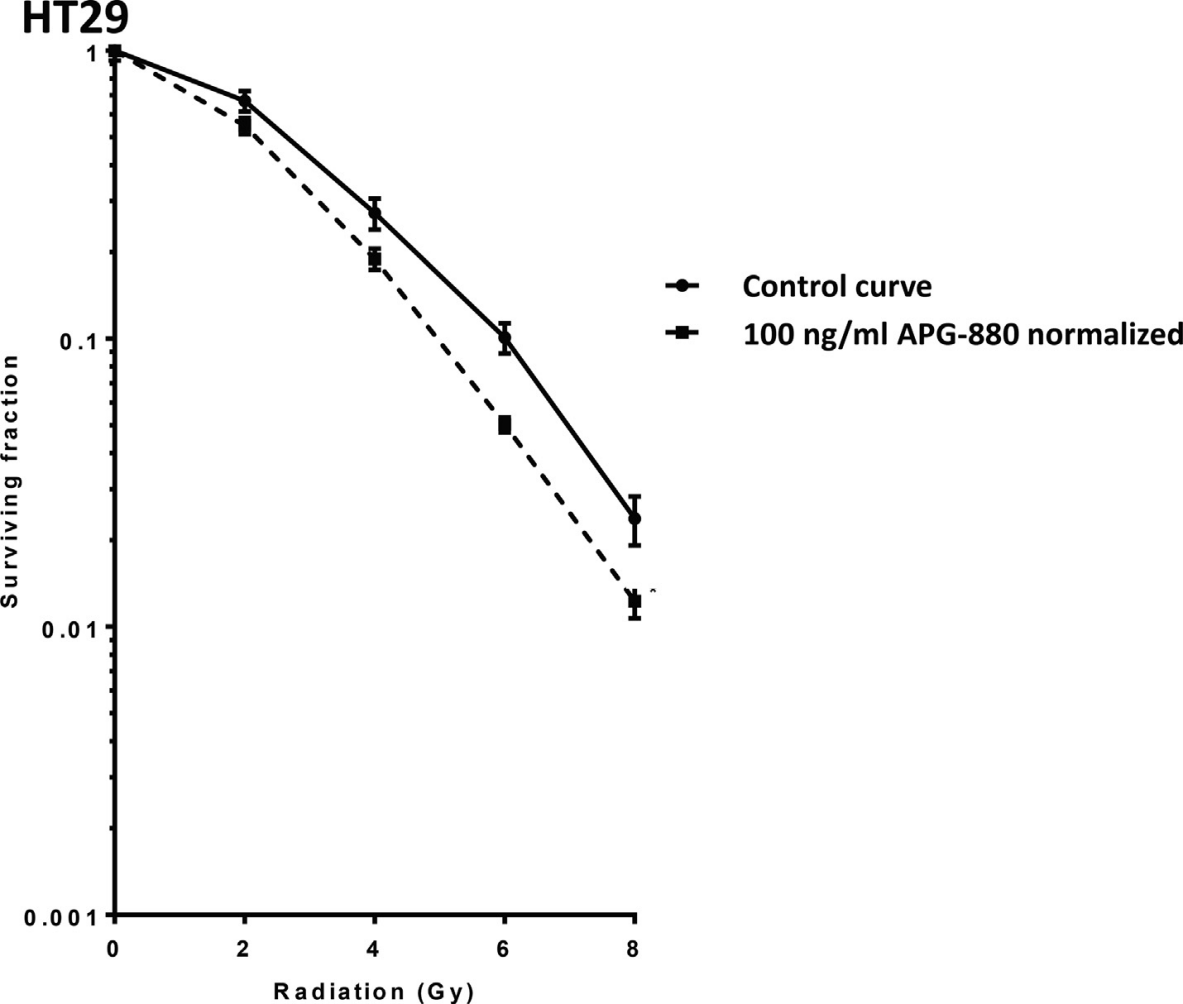
*APG-880 combined with radiation decreases clonogenic survival in HT29.* Clonogenic survival curves of HT29 cells in the absence (solid line) or presence of 100 ng APG-880 (normalized dashed line) are shown. Graphs show representative curves of 4 experiments in triplicate, error bars represent SEM. DEF37 = 1.3.

In clonogenic survival assays in which APG-880 and radiation were combined, HT29 displayed the most effective decrease in clonogenicity at doses around 100 ng/ml. Above these concentrations APG-880 alone was too cytotoxic and at lower concentrations no reduction in radiation-induced clonogenicity was seen. In Fig. 7 the combined results of 3 independent experiments in triplicate are shown for clonogenic assays with increasing doses of radiation combined with 100 ng/ml APG-880. This combination results in a Dose Enhancement Factor at 37% survival (DEF37) of 1.3. The HCT116 cell line on the other hand, was very sensitive to single modality treatment with APG-880 in the clonogenic survival assay, to an extent that at concentrations of APG-880 higher than 50 ng/ml only a fraction, i.e. 3%, of cells survived (Fig. 5B). That small fraction was considered non-representative and therefore no conclusive results on radiosensitization could be generated. Lower and less toxic concentrations, on the other hand, were ineffective to elicit a radiosensitizing effect. Due to this very small window, no dose range could be identified for combination experiments in HCT116 cells. These results are consistent with the higher sensitivity of HCT116 in cell death assays (Fig. 3, Fig. 4 and Fig. 5A) and the narrow dose range we found in HT29 cells. When we used a different readout than clonogenic survival, e.g. apoptosis, we did see an enhanced effect in both cell lines (Fig. 6A and B).

**Fig. 8:**
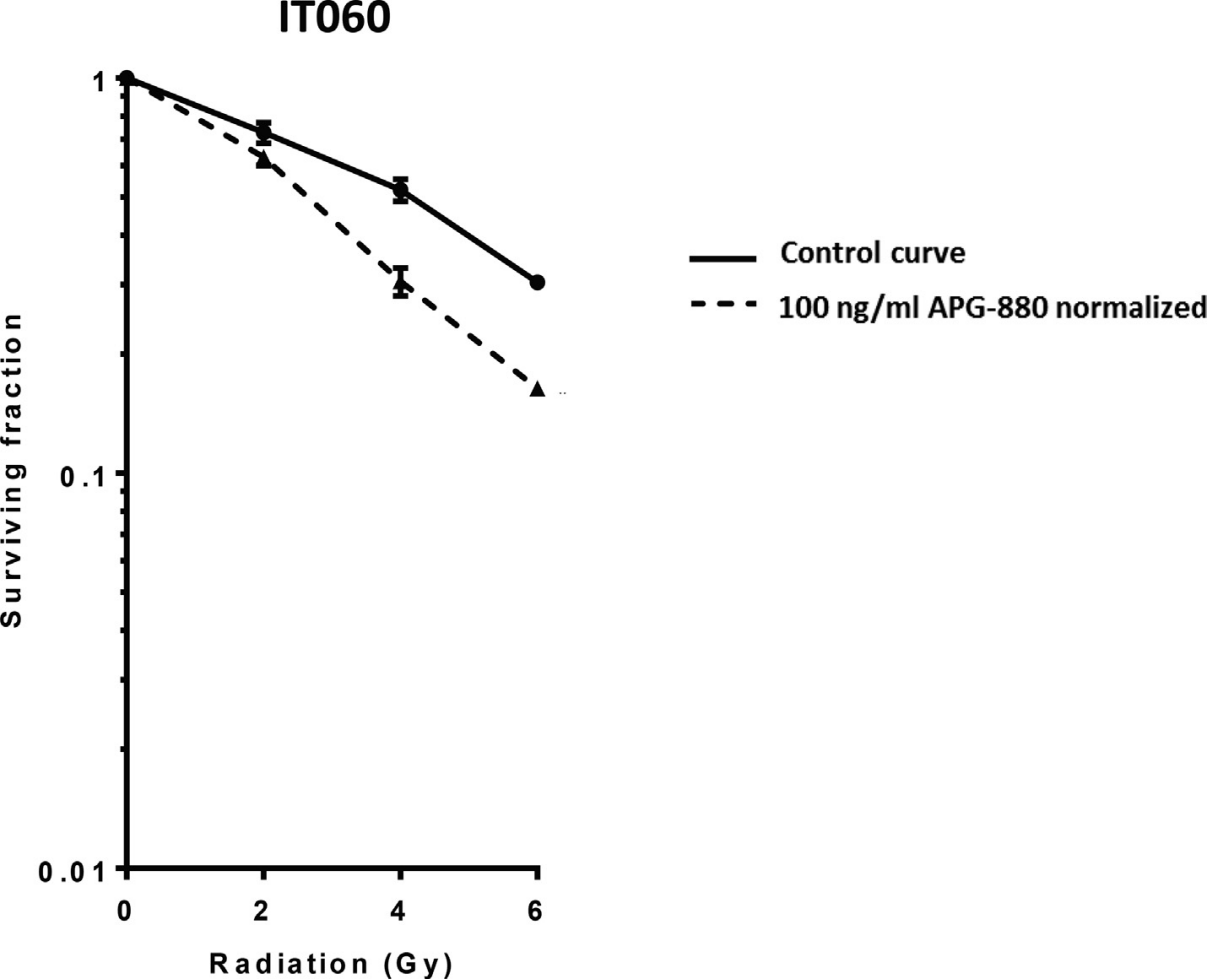
*APG-880 combined with radiation decreases clonogenic survival in organoids*. Clonogenic survival curves of colon carcinoma derived organoids ITO60 in the absence (solid line) or presence of 100 ng/ml APG-880 (normalized dashed line) are shown. Graphs show representative curves of 3 experiments in triplicate, error bars represent SEM. DEF37 = 1.5.

Based on DR4 and DR5 expression on organoids, and combined effects between APG-880 and radiation in CRC cell lines, we hypothesized that APG-880 could decrease clonogenic survival in CRC-derived organoids and enhance radiation responses. Indeed, the results in cell lines we tested the same concentration range and found that APG-880 reduces clonogenic survival after radiation, with a DEF_37_ of 1.5 (Fig. 8).

## 4 Discussion

In our search for a strategy to increase radiation efficacy, we describe here a novel combination of a second-generation TRAIL receptor agonist (APG-880) and radiation in a clinically relevant organoid model system.

We found that APG-880 and radiation show an enhanced combined effect in both short term and long term tumor cytotoxicity assays. The simultaneous activation of the intrinsic pathway by APG-880 and the extrinsic apoptotic pathway by radiation [21], [23] served as our hypothesis for a more than additive effect [22], [23]. Indeed, we found this interaction between both treatments in our apoptosis experiments as well as in our clonogenic assays.

The CRC organoid model presented here was selected for its known high TRAIL receptor expression level. As expected, under control culturing conditions the expression levels of both DR4 and DR5 receptors were readily detectable. Furthermore, the CRC model is a relevant model for drug-radiation combinations as colorectal tumors are frequently treated with radiation alone, or in combination with conventional chemotherapy.

One of the main limitations of the first-generation TRAs in the *in vivo* setting was the insufficient clustering and inefficient DISC formation by TRAIL antibodies which likely prohibited effective pro-apoptotic signaling [26], [28]. In addition, it has been shown that TRAIL-R antibodies have to compete with endogenous immunoglobulin G (IgG) for FcgR interaction at physiological concentrations [40]. Non-antibody TRAs that do not have these limitations like soluble human recombinant TRAs, are potent inducers of apoptosis but due to their short half-life, e.g. about 1 h for Dulanermin, and the fact that they also bind to the decoy receptors which attenuates their pro-apoptotic capacity, these recombinant TRAs have not been successful. Based on results of PK studies which show a half-life of more than one and a half days [41], and the hexavalent receptor clustering, the new second generation agonist also known as ABBV-621 is expected to perform better than the previously studied first generation TRAIL receptor agonists.

The tumor organoid models that we use here mimic more closely the heterogeneity and structural similarities of growing tumors [33], [42]. Therefore, organoids are considered to be a more representative model system for cancer research and an additional step between 2D in vitro lab research and preclinical animal research. Limited research has been done on radiation and organoids [43]. Here we show that patient derived CRC organoids are sensitive to radiation and that the apoptotic pathway plays an important role in this response. Furthermore, we show sensitivity towards the new drug APG-880 and the combination with radiation by two different assays. We tested radiosensitivity and combined effects with the cell titer glow 3D assay in three different patient derived organoids with different sensitivities towards both treatment modalities and all showed a combined effect.

Taken together, we demonstrate here that in short-term as well as long-term assays, radiation combined with APG-880 causes an enhanced effect on tumor cell kill in CRC cell lines and in CRC patient-derived organoids. Importantly, this clinically relevant new CRC organoid model system can be leveraged to address mechanistic combination studies with TRAIL-R agonists to better define the role of the apoptotic pathway to radiation treatment sensitivity.

## Declaration of Competing Interest

This work was partially supported by a research grant from AbbVie Inc. The funding source had no role in the study design. AbbVie Inc. participated in the interpretation of data, review, and approval of this publication.
